# Mitochondrial Base Editing: Recent Advances towards Therapeutic Opportunities

**DOI:** 10.3390/ijms24065798

**Published:** 2023-03-18

**Authors:** Bibekananda Kar, Santiago R. Castillo, Ankit Sabharwal, Karl J. Clark, Stephen C. Ekker

**Affiliations:** 1 Department of Biochemistry and Molecular Biology, Mayo Clinic, Rochester, MN 55905, USA; 2 Mayo Clinic Graduate School of Biomedical Sciences, Virology and Gene Therapy Track, Mayo Clinic, Rochester, MN 55905, USA

**Keywords:** mitochondria, mitochondrial DNA, heteroplasmy, base editing, DdCBE, TALED

## Abstract

Mitochondria are critical organelles that form networks within our cells, generate energy dynamically, contribute to diverse cell and organ function, and produce a variety of critical signaling molecules, such as cortisol. This intracellular microbiome can differ between cells, tissues, and organs. Mitochondria can change with disease, age, and in response to the environment. Single nucleotide variants in the circular genomes of human mitochondrial DNA are associated with many different life-threatening diseases. Mitochondrial DNA base editing tools have established novel disease models and represent a new possibility toward personalized gene therapies for the treatment of mtDNA-based disorders.

## 1. Introduction

Mitochondria are semi-autonomous organelles essential for the correct functioning of eukaryotic cells [1]. They are maternally inherited and generate the majority of the energy needed for cellular processes through ATP production [2]. Each mitochondrion contains multiple copies of mitochondrial DNA (mtDNA) in the mitochondrial matrix [1]. Human mtDNA is a circular, 16,569 bp-long, double-stranded DNA (dsDNA) molecule, typically presenting between 100 to 100,000 copies per cell [1]. Mammalian mtDNA is extremely compact, containing only one regulatory region [1]. Intergenic regions in mtDNA are largely absent, and mitochondrially encoded genes lack introns [1]. Human mtDNA encodes 2 rRNAs, 22 tRNAs, and 13 mRNAs required for the synthesis of 13 protein subunits of the mitochondrial respiratory complexes, which are essential for oxidative phosphorylation (OXPHOS) [1]. In particular, these 13 polypeptides constitute 7 of the 45 subunits of mitochondrial complex I, 1 of the 11 subunits of complex III, 3 of the 13 subunits of complex IV, and 2 of the 16 subunits of complex V [1,3]. The remaining protein subunits that comprise these respiratory complexes are encoded in the nuclear genome (nDNA) [4,5].

**Figure 1:**
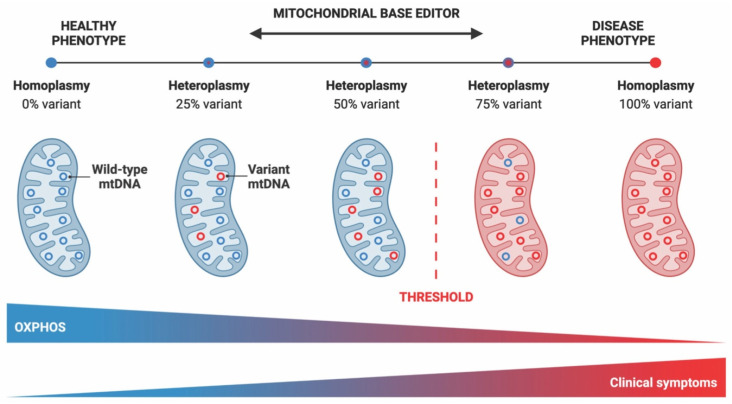
Heteroplasmy shifting with mitochondrial DNA base editors. The threshold of mtDNA heteroplasmy determines the onset of phenotypic manifestations. Generally, a threshold percentage of variant mtDNA must be surpassed for decreased OXPHOS and disease phenotypes. In general, the specific functional threshold depends on the mutation, cell type, and tissue type for a detectable phenotype. Utilizing programmable mitochondrial DNA base editors, heteroplasmy levels can be manipulated to create disease models or correct deleterious mutations. (This figure was created with BioRender.com, accessed on 15 January 2023).

Nuclear-mitochondrial genome interaction plays a key role in cellular metabolism [4,5], as over 1000 nuclear-encoded proteins contribute to oxidative phosphorylation, mtDNA maintenance and other mitochondrial functions [2,3,6]. Pathogenic mutations in either nDNA or mtDNA can cause mitochondrial diseases (MDs) [2,7,8], with a prevalence of ∼1 in 4300 in adults and ∼1 in 6700 in childhood [9,10]. Point mutations in any of the mitochondrially encoded genes that correspond to subunits of the electron transport chain, as well as in the genes encoding mitochondrial tRNAs and rRNAs, may lead to MDs [7,9]. When the proportion of harmful mtDNA variants reaches a critical level of heteroplasmy, meaning that both the pathogenic and wild-type mtDNA molecules coexist within the same cell or tissue, there can be biochemical defects that can give rise to disease (Figure 1) [11].

**Figure 2:**
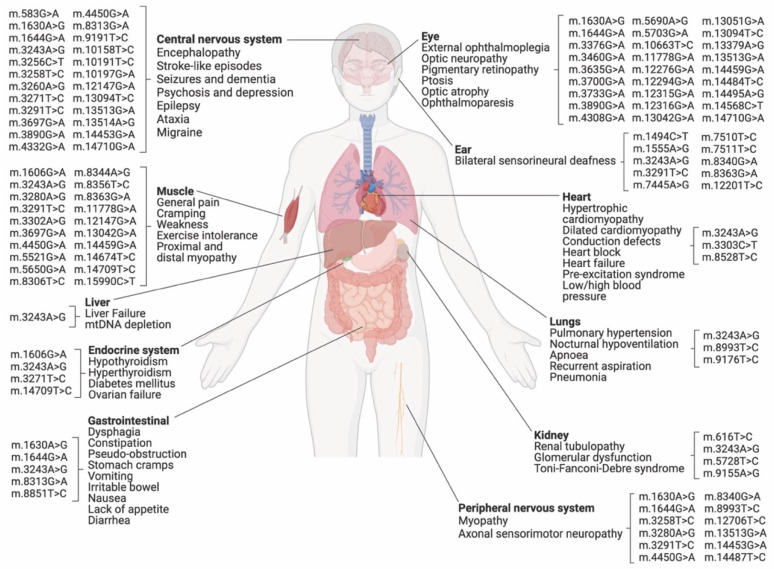
The potential use of present mitochondrial base editing technologies to correct or model disease-associated mtDNA point mutations. This figure displays common clinical features of mitochondrial disorders and their associated point mutations, as collated in Table 1 and derived from MITOMAP [13]. These mitochondrial diseases could potentially be corrected or modeled by C•G-to-T•A or A•T-to-G•C editing utilizing DbCBEs or TALEDs, respectively (Table 1). DdCBEs with evolved DddA6 show improved C•G-to-T•A editing at TC motifs, while DdCBEs with evolved DddA11 offer significant C•G-to-T•A editing at CC and AC along with TC motifs. Additionally, the recently-established DdCBE_Ss platform is highly effective at catalyzing C•G-to-T•A editing at GC motifs [14]. Moreover, the TALED platform enables A•T-to-G•C mtDNA editing in a context-independent manner, potentially increasing bystander editing at target loci. The existing base editing technologies hold promise for correcting or modeling pathogenic variants in mtDNA, with some limitations that need to be considered, as outlined above. (This figure was created with BioRender.com, accessed on 15 January 2023.)

Heteroplasmic conditions linked to point mutations can cause various clinical manifestations, including mitochondrial encephalomyopathy with lactic acidosis and stroke-like episodes (MELAS), Leigh syndrome, and myoclonic epilepsy with ragged red fibers (MERRF), among others [12] (Figure 2). MDs can occur at any age, from severe early-onset syndromes to milder late-onset conditions, and can affect several tissues in distinct manners [12]. Typically, organs with high energy demands, such as the brain and the heart, tend to be particularly affected by the presence of pathogenic mtDNA variants at high levels of heteroplasmy [12].

Primary mtDNA-based disorders are currently incurable [2]. These diseases often cause significant illness and can lead to premature death [2]. Moreover, variations in mtDNA can occur in individuals who are otherwise healthy and have been implicated in the etiology of age-related multifactorial diseases, including neurodegenerative disorders such as Parkinson’s disease, metabolic conditions, heart failure, and cancer [10,15]. The increasing prevalence of these conditions in our aging population highlights the need for the development of novel approaches for the investigation and prevention or treatment of these disorders [12,16].

Gene editing technologies can introduce targeted DNA modifications in cells or tissues to correct a genetic defect, and have already been successfully used to correct pathogenic mutations in the nuclear genome [17,18]. Mitochondrial gene therapy is a relatively new idea that, despite several challenges, has seen significant progress over the last few years [19]. Mitochondrial gene editing technologies can be designed to specifically act on variant mtDNA molecules, driving a heteroplasmic state toward a healthy, wild-type mtDNA population [20,21].

Broadly, two distinct modalities are currently used for mtDNA manipulation: (i) nuclease-based and (ii) base editing approaches. Conceptually, nuclease-based methods can be utilized to decrease the amount of variant mtDNA in mitochondria by specifically targeting and cleaving the mutant mtDNA molecules [22,23]. This technique relies on the premise that double-strand breaks (DSBs) in mtDNA induce the rapid degradation of the linearized molecule, instead of its repair [20,23]. If mutant mtDNA is specifically eliminated, the residual mtDNA, mostly wild-type, replicates and repopulates the organelle, resulting in the restoration of normal mtDNA levels. In particular, nuclease-based approaches have been described to include mitochondrially targeted restriction endonucleases (mitoREs), zinc-finger nucleases (mtZFNs), and transcription activator-like effector nucleases (mitoTALENs) [20,22,23,24,25,26]. These techniques have been extensively reviewed elsewhere [27,28,29,30]. Additionally, mitochondrially targeted CRISPR (mitoCRISPR) systems have also been reported [31,32,33,34]. However, these mitoCRISPR platforms have yet to be widely accepted within the scientific community due to the challenging nature of guide RNA (gRNA) import into the mitochondrial matrix, as well as a notable lack of follow-up studies [27,35,36].

Despite their potential usefulness for shifting heteroplasmy, mitochondrially targeted nucleases are unable to correct variant genomes [20,22]. Therefore, nuclease-based approaches cannot be utilized to rescue pathological conditions in homoplasmic states [20,28]. As mentioned above, the main alternative to nuclease-based strategies for mtDNA manipulation is base editing [37,38]. Consequently, in this work, we focused on recent advancements in the field of mitochondrial base editing, which holds the potential to treat diseases caused by pathogenic mtDNA point mutations in both heteroplasmic and homoplasmic contexts without the risk for mtDNA depletion [39,40]. This review also sought to provide insights into paths toward the development of therapeutic approaches for mtDNA-based disorders and the establishment of mitochondrial disease models to better understand the biology of these devastating diseases.

## 2. Mitochondrial Base Editing

In contrast to notable developments in nDNA manipulation since the repurposing of CRISPR as a gene editing platform [41,42], precise mtDNA editing was unfeasible until recently [39]. CRISPR-associated proteins transiently unwind dsDNA, enabling single-stranded DNA (ssDNA)-specific effector domains, such as APOBEC1 [43], to act locally on DNA sequences complementary to bases bound by the gRNA and to generate CRISPR base editors [37]. This key feature of CRISPR-associated proteins has been widely exploited for the precise editing of nDNA, utilizing ssDNA-specific deaminases as accessory effector domains [37,38]. Since a reproducible method for the efficient import of gRNAs into the mitochondrial matrix has remained elusive, CRISPR-based technologies cannot be effectively utilized for mtDNA editing [36]. However, other programmable DNA-binding proteins, such as TALEs, can be efficiently imported into mitochondria, although they do not intrinsically unwind dsDNA [44]. Therefore, mtDNA base editing was first accomplished using a novel protein capable of acting as a dsDNA-specific deaminase in conjunction with TALEs [45].

### 2.1. Mitochondrial Cytosine Base Editors

The discovery and characterization of double-stranded DNA deaminase A (DddA), a dsDNA-specific cytidine deaminase from *Burkholderia cenocepacia*, revolutionized the field of mtDNA editing [39]. Based on this enzyme, Liu lab [39] developed DddA-derived cytosine base editors (DdCBEs), the first agents capable of precise C-to-T editing in human mtDNA. Since their initial report, DdCBEs have been utilized for targeted mtDNA editing in mice [46], rats [47], zebrafish [48,49], plants [50], and human embryos [51,52], demonstrating their broad potential. In the following sections, we summarized major developments in mitochondrial cytosine base editing thus far, in chronological order. We envisioned that this compendium might facilitate the implementation and design of new technologies for mitochondrial gene editing.

#### 2.1.1. DddA-Derived Cytosine Base Editors (DdCBEs)

**Figure 3:**
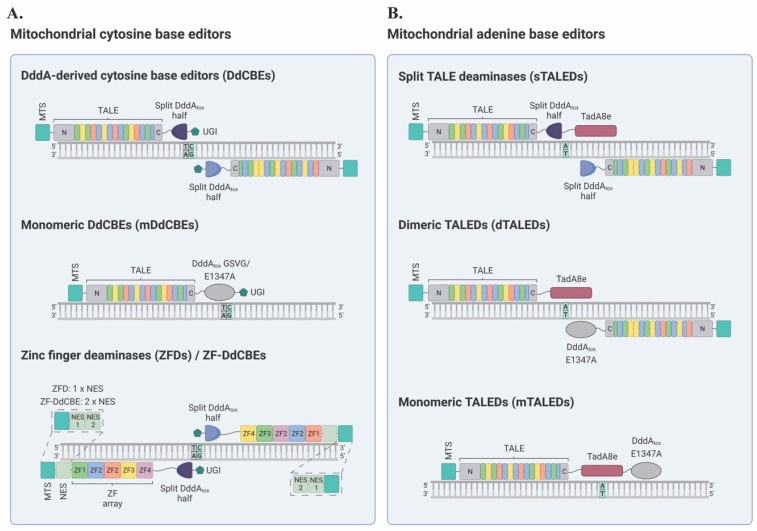
The mitochondrial DNA base editing toolbox. (**A**): Mitochondrial cytosine base editors. Target cytosines are shown in green, preceded by a 5′ thymine in gray. Top: DddA-derived cytosine base editors (DdCBEs). DddA_tox_ can be either G1333- or G1397-split. In addition to wild-type split DddA_tox_, variant DddA6 can be used for enhanced activity at TC targets, and variant DddA11 can be used for C-to-T editing at HC targets (H = A/C/T). Additionally, split DddA_tox_, in HiFi-DdCBEs incorporate the point modifications T1391A or K1389A. Alternative architectures, such as NES- or HHR-containing DdCBEs, are not illustrated. Middle: monomeric DdCBEs (mDdCBEs). DddA_tox_ GSVG/E1347A are full-length, nontoxic DddA_tox_ variants. Bottom: zinc finger deaminases (ZFDs)/ZF-DdCBEs (shown in a single panel and in a C-terminal + C-terminal configuration for simplicity). On top of the highlighted difference between the number of NES motifs, ZF-DdCBEs contain a 13-amino-acid linker between the ZF array and split DddA_tox_, whereas ZFDs contain a 24-amino-acid linker. Additionally, ZF-DdCBEs utilize variant G1397-split DddA_tox_ halves and engineered ZF scaffolds (details in text). (**B**): Mitochondrial adenine base editors. Target adenines are shown in green. Top: split TALE deaminases (TALEDs). Middle: dimeric TALEDs (dTALEDs). Bottom: monomeric TALEDs (mTALEDs). MTS: mitochondrial targeting signal; TALE: transcription activator-like effector; DddA_tox_: double-stranded DNA deaminase A toxin domain; UGI: uracil glycosylase inhibitor; NES: nuclear export signal; TadA8e: engineered deoxyadenosine deaminase variant. (This figure was created with BioRender.com, accessed on 15 January 2023.)

Canonical DdCBEs consist of two arms, each comprising a TALE protein fused to the N- or C-terminus of DddA_tox_ (the deaminase domain of DddA), followed by a uracil glycosylase inhibitor (UGI) (Figure 3A) [39]. Following well-established rules for the design of TALEs, these can be custom-made to target specific sequences within mtDNA [45,53,54]. The ensuing TALE-mtDNA interactions bring both arms of a DdCBE pair into close proximity, enabling the targeted reassembly of active DddA_tox_ [39]. Subsequently, cytosine residues in a 5′-TC context and within the spacer region, i.e., the sequence between the two TALE binding sites, are converted to uracil [39]. Provided that UGI impedes the excision of the resulting uracil residues, U•G intermediates are resolved into T•A base pairs during mtDNA replication [39], which takes place even in post-mitotic cells [55]. This process results in programmed C•G-to-T•A conversions in the mitochondrial genome [39].

In general, from the N-terminus to the C-terminus, the right-side halves of DdCBEs consist of a mitochondrial targeting signal (MTS) derived from COX8A, a FLAG tag, a TALE domain, a 2-amino-acid linker, a DddA_tox_ half, a 4-amino-acid linker, and UGI. Similarly, from the N-terminus to the C-terminus, the left-side halves of DdCBEs consist of an MTS derived from SOD2, an HA tag, a TALE domain, a 2-amino-acid linker, a DddA_tox_ half, a 4-amino-acid linker, and UGI (Figure 3A) [39]. The wild-type form of the deaminase domain DddA_tox_ is divided into two inactive halves to mitigate its toxicity [39]. In particular, DddA_tox_ can be split at the peptide bond between G1333 or G1397 and their respective following residues, resulting in distinct N- and C-terminal DddA_tox_ halves [39]. Therefore, there are four possible DdCBE configurations: left-G1333-C + right-G1333-N, left-G1333-N + right-G1333-C, left-G1397-C + right-G1397-N, and left-G1397-N + right-G1397-C, each of which can result in distinct editing patterns [39].

Regarding design recommendations, all DdCBE arms reported by Mok et al. [39] recognized 10–18 bp each and were separated by spacer regions of 14–18 bp. An additional design guideline suggested targeting sequences with a thymidine at the 3′ end, in addition to the 5′T rule for canonical TALEs [39,56]. Notably, certain DdCBE arms contained mismatched terminal TALE repeats, targeting T instead of G or C, which might lead to imperfect binding and negatively impact editing efficiencies [39]. Moreover, given that editing patterns can differ between DdCBE configurations, it has been recommended to test all four possible orientations for a particular target site [39]. Nonetheless, across five mtDNA genes, G1397-split DdCBEs preferentially edited TCs positioned 4–7 nucleotides upstream of the 3′ end of the spacer [39]. In contrast, G1333-split DdCBEs preferentially edited TCs positioned 4–10 nucleotides downstream of the 5′ end of the spacer [39]. Interestingly, cytosines in TCC contexts were also amenable to editing, often resulting in TCC-to-TTT conversions [39].

With regard to genome-wide target specificity, most DdCBE pairs tested were notably specific, inducing off-target editing at frequencies similar to those of untreated cells [39]. In contrast, a DdCBE pair with relatively short TALE-binding sequences, mismatched terminal TALE repeats, and a permissive right TALE N-terminal domain induced significant mtDNA off-target editing [39]. Moreover, DdCBE-induced nuclear off-target mutations were evaluated via targeted amplicon sequencing of three nuclear pseudogenes: *MTND6P4*, *MTND5P11*, and *MTND4P12* [39]. These sites were of interest, given that there were single nucleotide mismatches between either the left or right TALE-binding sequences of each DdCBE pair and its corresponding nuclear pseudogene [39]. Overall, only one statistically significant off-target editing event was detected, in *MTND6P4*, albeit at low frequencies (<0.2%) [39]. However, a nuclear genome-wide analysis might reveal additional off-target mutations [57].

#### 2.1.2. Zinc Finger Deaminases (ZFDs)

Following the development of DdCBEs [39], Lim et al. [58] engineered zinc finger deaminases (ZFDs). The main difference between DdCBEs and ZFDs lies on their respective DNA-binding moieties: ZFDs utilize ZF arrays instead of TALEs (Figure 3A) [58]. ZFDs were validated as programmable deaminases for both nuclear and mtDNA editing in HEK293T cells, with the latter being achieved using mitochondria-targeting ZFDs (mitoZFDs) [58]. In contrast to DdCBEs, in which split DddA_tox_ is invariably C-terminal, in mitoZFDs, split DddA_tox_ can be either C- or N-terminal [58]. Thus, a mitoZFD arm can be considered C-type or N-type depending on whether split DddA_tox_ is positioned at its C- or N-terminus [58]. In general, all mitoZFD constructs include the MTS from the human mitochondrial ATP synthase F1β subunit, a FLAG or an HA tag, a nuclear export signal (NES) from minute virus of mice (MVM), the N- or C-terminal half of DddA_tox_ split at G1397, a custom ZF array, and UGI (Figure 3A) [58].

In particular, from the N- to the C-terminus, a C-type arm consists of an MTS, a FLAG tag, an MVM NES, a ZF array, a 24-amino-acid linker, a DddA_tox_ half, a 4-amino-acid linker, and UGI (Figure 3A) [58]. Similarly, an N-type arm consists of an MTS, an HA tag, an NES, a DddA_tox_ half, a 24-amino-acid linker, a ZF array, a 4-amino-acid linker, and UGI [58]. Therefore, there are four possible mitoZFD architectures: CC (left C-type + right C-type), NC (left N-type + right C-type), CN (left C-type + right N-type), and NN (left N-type + right N-type) [58]. In the original mitoZFD report, the CC and CN configurations were tested across nine target sites in mtDNA, with higher editing efficiencies observed using mitoZFDs with the CN configuration [58]. However, a more thorough comparison between both architectures is still needed to determine if these observations are generalizable [58]. As expected, mitoZFDs edited cytosines in TC and TCC contexts [58]. Interestingly, two cytosines in an ACC context in the *MT-ND2* site were also significantly edited [58].

When designing mitoZFDs, each mitoZFD arm can be synthetized as an array of four zinc finger domains recognizing 12 bp each, separated by spacers of 7–15 bp [58]. Custom ZF arrays for de novo mitoZFDs can be designed and assembled using publicly available resources [58,59,60,61]. Furthermore, mitoZFD/DdCBE hybrid pairs can be used to generate distinct mutation patterns [58]. Interestingly, mitoZFDs, DdCBEs, and mitoZFD/DdCBE hybrid pairs with similar spacers often resulted in distinct editing patterns [58]. Concerning delivery, HEK293T cells were co-transfected with plasmids or mRNA encoding each arm of a mitoZFD or a mitoZFD/DdCBE hybrid pair [58]. Treated cells were harvested in bulk 4 days post-transfection, and targeted deep sequencing revealed mtDNA editing efficiencies of up to 30% [58].

Regarding mitochondrial genome-wide target specificity, mitoZFDs introduced hundreds of off-target edits at frequencies of >1% [58]. However, decreasing the dose of mRNA per monomer and introducing zinc finger modifications that reduced the affinity of the ZF array [62] resulted in high on-target activity with few detected mtDNA off-target edits [58]. Additionally, the nuclear off-target effects of mitoZFDs were evaluated by targeted amplicon sequencing of four nuclear sites with high sequence homology to two mitochondrial on-target sites [58]. The evaluated nuclear sequences differed from the mtDNA targets by 1–2 bp [58]. Interestingly, no off-target edits were detected at three nuclear sites with high sequence homology to the *MT-ND4L* on-target locus [58]. In contrast, off-target editing was observed at a nuclear region with high sequence homology to the *MT-ND2* on-target site, although at low frequencies (∼1%) [58].

To assess the stability of mitoZFD-induced mtDNA mutations, single cell-derived clonal populations were obtained from mitoZFD-treated HEK293T cells [58]. In general, most single cell-derived clones harbored mutations at frequencies similar to those of untreated cells [58]. However, base-edited single cell-derived clones had mutations with frequencies ranging from 26–98% [58]. These observations suggest that mitoZFDs introduce heteroplasmic mutations in a non-uniform manner in a group of cells [58].

#### 2.1.3. DddA6- and DddA11-Containing DdCBEs

Based on DdCBEs containing split wild-type DddA_tox_, Mok et al. [63] evolved DdCBE variants with enhanced activity and expanded targeting scope. To this end, phage-assisted continuous evolution (PACE) and phage-assisted non-continuous evolution (PANCE) were utilized. These are methods for the generation of improved biomolecules [64,65,66]. First, aiming to evolve DdCBEs with enhanced activity at TC targets, PANCE was performed, which resulted in the DddA_tox_ (T1380I) mutant, referred to as DddA1 [63]. Then, DddA1 was further evolved via PACE to obtain the variants DddA2 through DddA5 [63]. DdCBE pairs containing these variants showcased improved mtDNA editing efficiencies, particularly DddA5 [63]. Then, reasoning that mutation T1314I from DddA4 might promote reconstitution of split DddA_tox_ halves, this variation was incorporated into DddA5, resulting in DddA6 [63]. Overall, compared to canonical DdCBEs, DddA6-containing DdCBEs increased mtDNA editing frequencies at TC contexts by 3.3-fold on average [63].

Next, seeking to evolve DdCBEs capable of editing non-TC sequences, after a round of mutagenic drift, context-specific PANCE and PACE were used to generate the variants DddA7 through DddA11 [63]. These PACE-generated mutants displayed an expanded targeting scope in both bacterial plasmid assays and mtDNA editing experiments in vitro [63]. In particular, DddA11-containing DdCBEs significantly improved mtDNA editing efficiencies at TC, CC, and AC targets [63]. Nonetheless, within a single spacer with multiple editable substrates, these PACE-evolved variants displayed the highest mtDNA editing activity at TC contexts, followed by CC and AC contexts, in that order [63]. Interestingly, despite attempts to increase editing activity at GC sequences via PACE or PANCE, no DddA_tox_ variants that efficiently processed GC substrates in these bacterial assays were obtained [63]. However, DddA11-containing DdCBEs were capable of editing at GC targets in the *MT-ND4* site, albeit at low frequencies [63].

Regarding design recommendations, guidelines previously defined for DdCBEs remain valid [39], although spacers as short as 12 bp have been shown to support DdCBE-mediated editing in bacterial assays [63]. When utilizing traditional cloning-based methods for DdCBE generation, such as Golden Gate assembly [49,67], parental plasmids with the desired DddA_tox_ variant should be used. Notably, both DddA6 and DddA11 evolved from DddA_tox_ split at G1397 [63]. Thus, it might be expected that evolved DdCBEs containing the G1397 split would be more active than their G1333-split-containing counterparts [63]. Indeed, DddA6- and DddA11-containing DdCBEs in the G1333 split orientation induced lower on-target mtDNA editing compared to the G1397 orientation [63]. As for the editing windows, canonical and evolved DdCBEs displayed similar mutation patterns in artificial all-TC spacers, with DddA11 showcasing an overall larger editing window for spacers > 15 bp [63].

The mitochondrial genome-wide target specificity of evolved mitochondrial base editors was evaluated with DddA6- or DddA11-containing DdCBEs at two mtDNA target sites [63]. Overall, DdCBEs containing deaminase domains with higher activity and expanded targeting scope induced more mtDNA off-target edits compared to canonical DdCBEs [63]. It was also noted that likely promiscuous TALEs could negatively impact the specificity of a DdCBE pair, either canonical or evolved [63]. Interestingly, mitochondrial DdCBE-induced nuclear off-target mutations were not evaluated [63]. Instead, nuclear off-target editing, induced by nuclear-targeted DdCBEs with evolved DddA_tox_ variants, was analyzed [63]. The off-target prediction tool PROGNOS [68] was utilized to select a total of 19 nuclear off-target sites for two nuclear DdCBEs [63]. Overall, nuclear off-target editing remained similar between canonical and evolved DdCBEs, suggesting that DddA6 and DddA11 did not necessarily decrease DdCBE specificity in the nucleus [63].

Regarding delivery, HEK293T cells were co-transfected with plasmids encoding each arm of a DdCBE pair and harvested in bulk 3 days post-transfection [63]. Subsequently, targeted amplicon sequencing revealed targeted C•G-to-T•A conversions in mtDNA at frequencies of up to ∼30% in cells treated with either DddA6- or DddA11-containing DdCBEs [63]. For mtDNA editing in other human cell lines, the left and right arms of a DdCBE pair targeted to *MT-ND5* were fused to mCherry and eGFP, respectively, with a self-cleaving P2A sequence separating each arm from its corresponding fluorescent marker [63]. Then, cells were electroporated with each expression plasmid and sorted based on fluorescence 3 days after electroporation using fluorescence-activated cell sorting (FACS) to enrich for double-positive populations [63]. This approach significantly increased average editing levels—from less than 1% to 4–31% in HeLa cells, 11-fold in K562 cells, and 1.5-fold in U2OS cells, as determined via targeted amplicon sequencing [63].

Furthermore, DddA11-containing DdCBEs were utilized to introduce disease-associated mtDNA variants at non-TC positions in HEK293T cells [63]. Specifically, the missense m.11696G>A variant, associated with Leber’s hereditary optic neuropathy [69], and the nonsense m.13297G>A variant, implicated in renal oncocytoma [70], were introduced [63]. In sorted cells, these variants were induced at levels sufficient to alter mitochondrial function, which could have notable implications for mitochondrial disease modeling [63].

#### 2.1.4. Monomeric DdCBEs (mDdCBEs)

Despite being highly versatile technologies, a major limitation of DdCBEs [39] and mitoZFDs [58] is their dimeric architectures [71]. The requirement for two arms represents a synthesis bottleneck and complicates delivery strategies, especially for DdCBEs, which are significantly larger than mitoZFDs [39,58,71]. Hence, aiming to overcome these limitations, Kim lab [71] developed monomeric DdCBEs (mDdCBEs), which relied on non-toxic, full-length DddA_tox_ variants with reduced affinity for dsDNA. This mutagenesis-based approach represented an alternative to splitting DddA_tox_ into two inactive halves [39,71]. In addition to simplifying molecule manufacturing and delivery, mDdCBEs often produce distinct editing patterns compared to dimeric DdCBEs, broadening the scope of organellar genome editing [71]. Furthermore, given their simplified assembly compared to split DdCBEs, mDdCBEs might facilitate base editing screens, although bystander editing remains a reasonable concern [71].

To obtain a nontoxic, full-length DddA_tox_ variant that could support base editing, error-prone PCR was conducted to generate a library of DddA_tox_ variants [71]. These variants were then screened for deamination activity in vitro via Cas9-DddA_tox_ fusions directed to a site in the nuclear gene *TYRO3* in HEK293T cells [71]. By measuring cytosine conversion rates, this screening process led to the discovery of a nontoxic, full-length DddA_tox_ variant with four point modifications, termed the DddA_tox_ GSVG variant [71]. Subsequently, this mutant deaminase domain was fused to the C-terminus of TALE arrays designed to target mtDNA at different sites, resulting in mDdCBEs (Figure 3A) [71]. When transiently expressed in cultured human cells, mDdCBEs induced targeted C•G-to-T•A conversions in the mitochondrial genome at frequencies of up to 50% [71]. Thus, in terms of editing efficiencies, mDdCBEs were on par with their dimeric counterparts [71].

Overall, the architecture of an mDdCBE is almost identical to that of a single arm in a dimeric DdCBE pair, except that the deaminase domain corresponds to a full-length DddA_tox_ variant rather than a split DddA_tox_ half [71]. Interestingly, there were observations of mtDNA editing with mDdCBEs containing the active-site mutant DddA_tox_ E1347A [39] instead of DddA_tox_ GSVG, albeit less efficiently [71]. Hence, from the N- to the C-terminus, mDdCBEs consist of the SOD2 or COX8A MTS, an HA or FLAG tag, a TALE domain, a 2-amino-acid linker, DddA_tox_ GSVG or E1347A, a 4-amino-acid linker, and UGI (Figure 3A) [71]. Regarding mDdCBE design guidelines, similar to their canonical and dimeric counterparts [39], mDdCBEs can only edit cytosines in TC contexts [71]. Moreover, mDdCBEs preferentially edit TCs positioned 4–11 nucleotides downstream of a TALE-binding sequence [71]. However, mDdCBEs might also induce bystander editing as far as 61 bp downstream of the 5′ end of a TALE-binding site [71].

Throughout the study, mDdCBEs were synthesized with the same TALE arrays as those used for dimeric DdCBEs [71]. As a result, there were four possible mDdCBE formats, termed L/R-GSVG/E1347A [71]. Genome-wide target specificity was evaluated utilizing all four mDdCBE formats targeted to the *MT-ND1* and *MT-ND6* sites, along with dimeric DdCBEs and TALE-free split or full-length DddA_tox_ variants as controls [71]. At the *MT-ND1* locus, all mDdCBEs were less specific than dimeric DdCBEs, particularly L-GSVG [71]. In contrast, at the *MT-ND6* site, mDdCBEs and dimeric DdCBEs displayed similar off-target editing, with L-E1347A and R-E1347A being more specific than dimeric DdCBEs [71]. These results suggested that the specificity of mDdCBEs was not necessarily worse than that of split DdCBE pairs [71]. Interestingly, transfection of mDdCBE-encoding mRNA, instead of plasmid DNA, resulted in lower off-target editing, concomitant with reduced on-target editing efficiencies [71].

Moreover, the nuclear off-target mutations induced by mDdCBEs were evaluated by targeted amplicon sequencing of a single nuclear pseudogene, *MTND4P12*, which had high sequence homology with the *MT-ND4* on-target site. Notably, no mDdCBE-induced off-target editing was detected at this nuclear pseudogene [71]. However, a nuclear genome-wide specificity evaluation might reveal several off-target editing events [57].

In terms of delivery, HEK293T cells were transfected with mDdCBE-encoding plasmid or mRNA [71]. Treated cells were harvested in bulk 3 days post-transfection for on-target editing analysis via targeted amplicon sequencing [71]. Additionally, HEK293T cells were transduced with single mDdCBE-encoding AAV2 vectors at multiplicities of infection ranging from 10,000 to 500,000 [71]. Then, treated cells were collected in bulk 6 days post-transduction and evaluated for AAV-mediated base editing, which reached efficiencies as high as 99.1% at the *MT-ND4* site, and 59.8% at the *MT-ND1* site [71]. These results demonstrated that, unlike split DdCBEs, mDdCBEs could be packaged and delivered via single recombinant AAV vectors in vitro [71]. Additionally, these observations showed that nearly homoplasmic mtDNA mutations (>99%) could be obtained in cultured human cells via AAV-mediated base editing [71].

#### 2.1.5. High-Fidelity DdCBEs (HiFi-DdCBEs)

Evaluations of the mitochondrial genome-wide target specificity of mtDNA base editing technologies have consistently shown measurable off-target activities in the nucleus [39,58,63,71]. Indeed, conventional DdCBEs [39], evolved DdCBEs [63], mDdCBEs [71], and mitoZFDs [58] can each induce off-target mutations in the mitochondrial genome. Moreover, a comprehensive evaluation of the nuclear genome-wide target specificity of canonical DdCBEs demonstrated that these base editors induced several nuclear off-target mutations in vitro [57]. In contrast, analyses of nuclear off-target editing by enhanced DdCBEs [39], mDdCBEs [71], and mitoZFDs [58] have remained limited to targeted amplicon sequencing of a few predicted off-target sites. More fully assessing the levels and significance of these off-target edits is an important next step in the field, both in mtDNA and nDNA [39,57,58,63,71,72].

Excessive imprecision of mtDNA base editing technologies has the potential to limit their use for disease modeling and therapeutic applications. Therefore, aiming to circumvent this constraint, Kim lab [72] developed high-fidelity DdCBEs (HiFi-DdCBEs), which relied on interface-engineered split DddA_tox_ variants. Wild-type split DddA_tox_ halves can spontaneously reassemble independently of TALE-DNA interactions, leading to unwanted off-target mutations in nDNA or mtDNA [57,72]. To address this issue, amino acid residues within the interface between split DddA_tox_ halves were substituted with alanine, which has a chemically inert and non-bulky side chain [72]. These alanine substitutions resulted in split DddA_tox_ variants that required DNA binding of their respective TALE arrays in order to reassemble and form a functional deaminase domain [72]. Remarkably, HiFi-DdCBEs displayed on-target editing efficiencies on par with conventional DdCBEs while avoiding hundreds of off-target edits in human mtDNA [72].

There are two recommended split DddA_tox_ variants for HiFi-DdCBEs [72]: T1391A and K1389A. Both are functional in either the G1397- or G1333-split configurations [72]. In general, the T1391A variant is recommended for use to achieve the highest specificities, although HiFi-DdCBEs with this variant might not be as active as canonical DdCBEs [72]. Likewise, the K1389A variant is recommended when high activity is preferred over high specificity [72]. Notably, these variants can be introduced in DddA6 and DddA11 to generate HiFi-DdCBEs with enhanced activity and expanded targeting scope [63,72]. However, since DddA6 and DddA11 evolved from G1397-split DddA_tox_ [63], it is reasonable to adhere to this configuration when designing DddA6- and DddA11-containing HiFi-DdCBEs [72]. Moreover, general design guidelines, previously defined for DdCBEs, have remained valid, i.e., TALE-binding sequences between 10–18 bp separated by spacer regions of 12–18 bp in length [39,63].

The off-target editing induced by HiFi-DdCBEs in the mitochondrial genome was thoroughly evaluated [72]. For example, a wild-type G1397-split DdCBE pair targeted to the *MT-ND1* site induced 238 off-target edits in mtDNA at frequencies of ≥1.0% [72]. In contrast, the corresponding K1389A-containing HiFi-DdCBE pair induced 5 off-target edits, and the T1391A-containing pair avoided mtDNA off-target editing altogether [72]. The on-target editing efficiencies induced by HiFi-DdCBEs at the *MT-ND1* site were noted to be on par with those displayed by canonical DdCBEs [72]. In general, similar specificity profiles were observed across four different regions in human mtDNA [72]. In terms of nuclear off-target editing, in contrast with canonical DdCBEs, HiFi-DdCBEs avoided TALE-dependent off-target editing in three nuclear pseudogenes and TALE-independent off-target editing in the nuclear genome at five candidate sites, as reported by Lei et al. [57,72].

Concerning delivery, for all experiments, HEK293T cells were transfected with DdCBE-encoding plasmids at a dose of 500 ng per plasmid [72]. Subsequently, cells were harvested 4 days post-transfection and genomic DNA was purified. Then, targeted amplicon sequencing was utilized for mtDNA on-target editing and nDNA off-target editing analyses [72]. Similarly, whole mitochondrial genome sequencing was used for mtDNA off-target editing evaluations [72].

#### 2.1.6. Zinc Finger DdCBEs (ZF-DdCBEs)

Based on the development of DdCBEs [39], Willis et al. [73] engineered zinc finger DdCBEs (ZF-DdCBEs). Similar to mitoZFDs [58], ZF-DdCBEs utilize ZF arrays instead of TALEs as DNA-binding moieties (Figure 3A). However, optimization of mitoZFDs was limited to varying the length of the linker between the ZF arrays and split DddA_tox_ halves, testing different spacer lengths, and domain ordering [58]. In contrast, optimization of ZF-DdCBEs was far more comprehensive [73]. In particular, the following additional aspects were evaluated: mitochondrial import, nuclear export, residual cellular uracil-DNA glycosylase activity, ZF array length and scaffold engineering, and enhancement of DddA_tox_ activity [73]. Notably, side-by-side comparisons between mitoZFDs and ZF-DdCBEs across multiple sites in mtDNA suggested that ZF-DdCBEs consistently edited on-target sites at higher frequencies than mitoZFDs [73]. Moreover, several strategies for the development of ZF-DdCBE variants with enhanced specificities were explored [73].

First, the N- and C-terminal fragments of DddA_tox_ in the G1397-split format were incrementally truncated, and the resulting variants were tested for on- and off-target editing in different combinations [73]. This strategy resulted in modest improvements in specificity [73]. Then, scanning mutagenesis efforts revealed point mutations in the C-terminal fragment of G1397-split DddA_tox_ that weakened the association between split DddA_tox_ halves, increasing ZF-DdCBE specificity [73]. Additionally, ZF-DdCBE variants with increased negative charge at the termini of DddA_tox_ displayed moderate enhancements in specificity compared to canonical pairs while maintaining high on-target editing [73]. Furthermore, adding a catalytically inactivated N-terminal fragment of G1397-split DddA_tox_ downstream of its C-terminal fragment significantly increased ZF-DdCBE specificity relative to a canonical pair. Finally, these strategies were tested in tandem, producing five high-specificity (HS) ZF-DdCBE variants [73].

The optimization of the ZF-DdCBE architecture, combined with the development of versions with increased specificities, led to the configurations v8^HS1^ to v8^HS5^, which were validated across mtDNA [73]. As expected, v8^HS^ ZF-DdCBEs consistently resulted in reduced off-target editing compared to all preceding variants [73]. However, the observed improvements in specificity were often concomitant with moderate reductions in activity [73]. Nonetheless, some v8^HS^ ZF-DdCBEs showcased both increased specificity and enhanced on-target editing [73]. In detail, HS1 was defined by the single point modification N18K in DddA_tox_. Similarly, HS2 contained two point modifications: N18K and P25A. Likewise, HS3 incorporated N18K and P25K. Comparably, in addition to both the N18K and P25A point modifications, HS4 contained an N-terminal fragment of G1397-split DddA_tox_ that was C-terminally truncated by 3 amino acids [73]. Finally, HS5 differed from HS4 in that it contained the P25K single point modification, instead of P25A [73].

In addition to engineering high-specificity variants by modifying DddA_tox_, a set of ZF scaffolds for improved ZF-DdCBE performance was defined, namely: X1, AGKS, V2, and V20 [73]. In general, a ZF scaffold can be defined as a beta-motif, an alpha-motif, and a flexible linker motif [73]. Typically, the sequences of these motifs vary between the zinc fingers within a ZF array [73]. Thus, aiming to create high-performance ZF scaffolds, ZF-DdCBEs containing ZF arrays with identical repeating scaffolds were developed [73]. In this strategy, sequence variability was limited to the DNA-binding residues within each individual ZF [73]. The X1 scaffold was derived from canonical *ZNF268*-like ZFs [73]. Similarly, the AGKS scaffold was derived from the human transcription factor Sp1C [73]. Moreover, the V2 and V20 scaffolds were derived from a human proteome-wide analysis [73]. In terms of on-target editing, ZF-DdCBE pairs with the novel ZF scaffolds consistently outperformed canonical ZF-DdCBEs [73].

Notably, as in mitoZFDs [58], split DddA_tox_ halves in ZF-DdCBEs can be either N- or C-terminally fused to a ZF array [73]. Thus, ZF-DdCBEs can be designed in four different configurations: C-terminal + C-terminal, C-terminal + N-terminal, N-terminal + C-terminal, or N-terminal + N-terminal [73]. Specifically, from the N- to the C-terminus, a C-terminal v8 ZF-DdCBE arm consists of the following domains: an MTS from the human *ATP5F1B* gene, a FLAG tag, an MVM NES, a 2-amino-acid linker, a MAPKK NES, a 2-amino-acid linker, an enhanced ZF array, a 13-amino-acid linker, a variant G1397-split DddA_tox_ half, a 4-amino-acid linker, and UGI (Figure 3A) [73]. For the ZF arrays, any of the previously defined ZF scaffolds can be used (i.e., X1, AGKS, V2, or V20). Additionally, the variant G1397-split DddA_tox_ has the point modifications T1380I, E1396K, and T1413I [63,73]. Additionally, to minimize off-target activity while maintaining high on-target editing, variants HS1-HS5 can be incorporated into a v8 ZF-DdCBE pair (obtaining a v8^HS^ pair) [73].

ZF-DdCBE design guidelines for mtDNA editing recommend using the v8 architecture and ZF scaffold X1 [73]. For ZF array length, it has been recommended to start with a 3ZF + 3ZF configuration, i.e., ZF arrays composed of three ZFs each [73]. Since a single ZF recognizes 3 bp, the minimum recommended length of a ZF array-binding sequence in a ZF-DdCBE is 9 bp [73]. With respect to spacer length, spacing regions between 4–20 bp were shown to support ZF-DdCBE-mediated mtDNA editing in vitro [73]. Moreover, for a given ZF-DdCBE pair, it has been recommended to test both split DddA_tox_ orientations [73]. Once an efficient ZF-DdCBE pair has been identified, it can be further optimized by varying the lengths of the ZF arrays to up to 6 ZFs each, as well as by testing the ZF scaffolds AGKS, V2, and V20 [73]. Furthermore, as stated above, if specificity is critical, the HS1-HS5 variants can be incorporated into the optimized v8 ZF-DdCBE pair [73].

Interestingly, an optimized 3ZF + 3ZF ZF-DdCBE pair with the AGKS scaffold was able to install the m.8340G>A pathogenic variant within *MT-TK* in HEK293T cells with an efficiency of 31% [73]. Similarly, an optimized 5ZF + 5ZF ZF-DdCBE pair with the AGKS scaffold introduced the missense m.3177G>A mutation within *Nd1* in mouse C2C12 cells, with an efficiency of 39% [73]. Moreover, optimized v8^HS1^ ZF-DdCBE pairs were packaged into single AAV2/9 vectors in order to facilitate in vivo mtDNA base editing in newborn P1 mice [73]. Each ZF-DdCBE pair was expressed under a single CMV promoter, with each arm separated by a skipping P2A peptide [73]. This strategy was successful in inducing the m.7743G>A or m.3177G>A variants in the heart, liver, and quadriceps skeletal muscle of injected mice, with efficiencies between 12–83% [73]. These results demonstrated that ZF-DdCBEs could be delivered via single AAV vectors [73]. However, mitochondrial off-target editing remains a concern for in vivo applications [73].

In conclusion, comprehensively optimized ZF-DdCBEs were developed [73]. These constructs functioned as compact mitochondrial base editors that facilitated base editing both in vitro and in vivo, the latter being achievable via single recombinant AAV vectors [73]. Nonetheless, despite their versatility, a major concern of ZF-DdCBEs is their potential off-target activity [73]. It should be noted that the off-target effects of ZF-DdCBEs have been evaluated using amplicon-wide analyses, rather than genome-wide surveys of the mitochondrial genome [73]. In addition, the nuclear off-target mutations induced by mitochondrially targeted ZF-DdCBEs have not yet been reported [73]. In general, regardless of their significantly larger size, DdCBEs continue to be more specific and precise than ZF-DdCBEs [73]. Moreover, efficient ZF-DdCBE design and optimization remain a bottleneck that could hinder the widespread adoption of this technology within the broader scientific community.

#### 2.1.7. Additional Strategies to Limit DdCBE-Induced Off-Target Mutagenesis

To better understand the genome-wide specificity of DdCBEs, Lei et al. [57] conducted a comprehensive examination of the nuclear off-target effects induced by DdCBEs in cultured human cells. An unbiased analysis of the DdCBE editome revealed hundreds of nDNA off-target sites, which were classified as either TALE array sequence (TAS)-dependent or TAS-independent [57]. TAS-dependent off-target sites have high sequence homology (with no more than three mismatches) to on-target TALE-binding sequences, and are typically determined by a single DdCBE arm [57]. In contrast, TAS-independent off-target sites do not share high sequence similarity with on-target TALE-binding sequences and can be universally induced by different DdCBEs [57]. These findings, along with the more widely characterized DdCBE-induced mitochondrial genome-wide off-target effects [39,63,72], highlighted the need for highly specific mtDNA base editors [57]. To this end, alternative strategies have been reported with varied outcomes [58,71,72,73].

Lei et al. [57] tested three strategies for increasing the specificity of DdCBEs. The first strategy involved adding an NES downstream of either TALE, split DddA_tox_, or UGI to reduce the undesired nuclear localization of DdCBE pairs [57]. These modified DdCBEs supported high on-target mtDNA editing with significant reductions in nuclear off-target effects in HEK293T cells [57]. The second approach involved co-expressing nuclear-targeted DddI_A_ with canonical DdCBEs to inhibit their unwanted activity on nDNA [57]. DddI_A_ is a naturally occurring protein that occludes the active site of DddA_tox_ [39]. This strategy resulted in a significant reduction in nuclear off-target effects and, interestingly, a mild decrease in mtDNA on-target editing [57]. The third strategy involved incorporating rationally selected mutations into DddA_tox_ to decrease its DNA binding affinity [57]. A global off-target effect analysis of these approaches revealed that adding NES downstream of UGI and DddI_A_ co-expression were the most effective strategies in preventing nDNA off-target mutagenesis [57].

Lee et al. [74] tested the on-target activity and nuclear genome-wide specificity of DdCBEs containing an NES (termed DdCBE-NES) in mouse embryos. Similar to the modified DdCBEs tested by Lei et al. [57], the DdCBE-NES pairs contained an NES downstream of UGI [74]. To evaluate this strategy, a DdCBE-NES pair, designed to introduce the m.12918G>A variant within *Nd5*, was microinjected as mRNA into mouse zygotes, which were then cultured to the blastocyst stage [74]. The specificity of DdCBE-NES was initially evaluated at one potential TAS-dependent nuclear off-target site, which showed a significant improvement compared to canonical DdCBEs [74]. Additionally, whole-genome sequencing analysis of m12918G>A-harboring pups revealed that DdCBE-NES induced far fewer nuclear off-target mutations than canonical DdCBEs [74]. Furthermore, DdCBE-NES achieved higher on-target editing than DdCBEs, suggesting that NES addition could both decrease nDNA off-target editing and increase mtDNA on-target activity [74].

In addition to installing the m.12918G>A mutation within *Nd5* via DdCBE-NES, Lee et al. [74] compared DdCBE-NES and DdCBEs in the *TrnA* and *Rnr2* sites in mouse embryos. On average, DdCBE-NES resulted in higher on-target editing efficiencies than canonical DdCBEs at both target regions [74]. Interestingly, neither DdCBE-NES or DdCBEs installed undesired mutations at single potential TAS-dependent nuclear off-target sites [74]. No additional nuclear genome-wide analyses were conducted [74]. Notably, a strategy, combining wild-type mtDNA-specific mitoTALENs and DdCBE-NES or DdCBEs, was evaluated [74]. However, this alternative approach, which was shown to be somewhat effective, was focused on improving DdCBE on-target activity rather than specificity [74]. It is also worth noting that three mice, harboring the m.12918G>A variant at low heteroplasmy levels, developed a hunchback phenotype between 2–8 weeks of age and died within a week of the outbreak [74].

In their report of a DdCBE library for the ablation of all protein-coding genes in mouse mtDNA, Minczuk lab [75] tested three approaches aimed at limiting off-target mutagenesis. These strategies were based on the hypothesis that decreasing DdCBE expression levels was concomitant with reductions in off-target activity [75]. First, a 3′K19 hammerhead ribozyme (HHR) [76] was added downstream of the stop codon of DdCBE monomers, resulting in DdCBE-coding mRNA transcripts that were susceptible to degradation [75]. Alternatively, DdCBE pairs were encoded in single plasmids, with each arm separated by a T2A element, which was expected to result in decreased expression of the downstream monomer [75,77]. Finally, both strategies were combined, generating HHR-equipped and T2A-linked DdCBEs, in which both arms were separated by a T2A element, and the right arm was followed by a 3′K19 HHR [75]. In these approaches, all plasmids co-expressed DdCBE monomers and eGFP or mCherry, enabling enrichment via FACS [75].

As hypothesized, the modified DdCBEs led to significant reductions in mtDNA off-target mutagenesis, as compared to canonical DdCBEs in vitro [75]. Additionally, as expected, increased specificity was often accompanied by reduced on-target activity [75]. In general, the combined strategy resulted in the most notable improvements in on-target/off-target ratios, bringing off-target editing in mtDNA down to background levels [75]. Furthermore, nuclear genome-wide specificity was evaluated in NIH/3T3 cells, transfected with either a canonical *Atp6*-specific DdCBE pair or a corresponding T2A-linked construct [75]. Whole-genome sequencing revealed 109 potential off-targets sites, one of which showed an increased proportion of T•A in C•G positions in cells treated with canonical DdCBEs, but not with the T2A-linked constructs [75]. Notably, near-homoplasmic mtDNA variants were installed in NIH/3T3 cells with high specificity via the stable expression of either T2A-linked or T2A-linked and HHR-equipped DdCBEs [75].

In summary, several strategies have been reported for limiting DdCBE-induced nuclear and mitochondrial off-target mutagenesis. These include NES addition [57,74], DddI_A_ co-expression [57], incorporation of DddA_tox_ variants with reduced binding affinity [57], and HHR-equipped and/or T2A-linked DdCBEs [75]. Other strategies include dose titration [71], split-dimer interface engineering [72], truncation of split DddA_tox_ halves [73], and incorporation of split variant DddA_tox_ halves with weakened associations [73]. Thus far, to the best of our knowledge, no comprehensive comparison between these strategies has been reported. Typically, novel tools and their properties are compared with canonical DdCBEs, and strategies aimed at limiting off-target mutagenesis are contrasted with a subset of approaches. Therefore, researchers must carefully select the base editor that best suits their needs, including the incorporation of a strategy for the limitation of off-target activity.

### 2.2. Mitochondrial Adenine Base Editors

Mougous lab [39] made a groundbreaking discovery in the field of genome editing with the identification of the DddA_tox_ protein. This unique nucleic acid deaminase proved to be capable of accepting dsDNA as a substrate, thus catalyzing cytidine deamination as an intrinsic activity—a combination of biochemical properties that had not been previously observed [39]. Mok et al. [39] reported on two significant aspects of DdCBEs in relation to DddA_tox_. First, the intrinsic deaminase activity of DddA_tox_ could be significantly impaired by mutating the active site (E1347A). Second, the fusion of a UGI molecule downstream of DddA_tox_ led to a marked increase in net C-to-T editing efficiencies from this intrinsic cytidine deaminase, and a reduction in indel byproducts [39].

Leveraging the DdCBE technology, Kim lab [40] developed the first TALE deaminases (TALEDs) with the ability to introduce A-to-G substitutions (equivalent to T-to-C in the opposite strand) in mtDNA. Broadly, these A-to-G base editors had four fundamental components: (1) an N-terminal MTS, (2) a TALE designed to bind a specific DNA sequence, (3) split DddA_tox_ or full-length DddA_tox_ E1347A as potential dsDNA unwinders, and (4) TadA8e, an ssDNA-specific adenine deaminase variant enzyme derived from *E. Coli* (Figure 3B). In particular, three main architectures of TALEDs were engineered for A-to-G editing in mtDNA: split TALEDs (sTALEDs), dimeric TALEDs (dTALEDs), and monomeric TALEDs (mTALEDs) (Figure 3B) [40].

Initial experiments showed around 1% editing in the *MT-ND1* and *MT-ND4* loci in HEK293T cells using a TALE-TadA8e fusion protein without DddA_tox_ [40]. TadA8e is known to deaminate adenine in ssDNA with remarkable efficiency [78]. However, without local dsDNA unwinding, TadA8e cannot efficiently catalyze A-to-G conversions on mtDNA [40]. Based on these results, DddA_tox_ was utilized in a series of designs that facilitated efficient mitochondrial A-to-G editing [40]. Although the exact mechanism of action was not determined, it is reasonable to hypothesize that DddA_tox_ can function as a dsDNA unwinder, providing an accessible ssDNA substrate to TadA8e [40]. The first design, termed sTALED, similar to the canonical DdCBE architecture, used split DddA_tox_ halves [40]. However, to avoid C-to-T editing, UGI was removed [40]. Then, to facilitate A-to-G conversions, TadA8e was C-terminally fused to a single arm within the sTALED pair [40]. The resulting architecture displayed significant levels of targeted A-to-G editing in mtDNA [40]. Notably, keeping one copy of UGI fused to the sTALED arm without the TadA8e enzyme, which was kept in the opposite arm, led to the simultaneous introduction of targeted C-to-T and A-to-G conversions within spacer regions [40].

Subsequently, Kim lab [40] modified the original TALED design to develop the alternative dTALED and mTALED architectures [40]. For the dTALED architecture, similar to sTALEDs, two TALE proteins were used in a tail-to-tail configuration to target a desired DNA sequence [40]. Each TALE was fused to either TadA8e or full-length DddA_tox_ E1347A [40] (Figure 3B). For the mTALED format, full-length DddA_tox_ E1347A was C-terminally fused to TadA8e, downstream of a mitochondrially targeted TALE, resulting in the MTS-TALE-TadA8e-DddA_tox_ E1347A architecture [40] (Figure 3B). Overall, mTALEDs showed significant A-to-G editing within target spacer regions [40]. Additionally, side-by-side comparisons between the MTS-TALE-TadA8e-DddA_tox_ E1347A architecture and an alternative MTS-TALE-DddA_tox_ E1347A-TadA8e design showed that the former showcased better editing efficiencies than the latter [40].

To compare the editing efficiencies of these three programmable deaminases, the authors targeted 12 different sites in human mtDNA with sTALEDs, mTALEDs, and dTALEDs [40]. It was observed that, overall, sTALEDs performed better than mTALEDs and dTALEDs [40]. Nonetheless, mTALEDs and dTALEDs induced higher editing efficiencies than sTALEDs at specific target sites [40]. It was also noted that sTALEDs, mTALEDs, and dTALEDs, targeted to the same spacer region, produced distinct editing patterns, widening the scope of mitochondrial genome editing [40]. Of note, Kim lab [79] recently reported targeted editing of chloroplast DNA in *Lactuca sativa* utilizing TALEDs, thus demonstrating the adaptability of this system.

These various TALED architectures demonstrated a dual functionality for the DddA_tox_ protein [40]. In particular, the outcomes of editing with dTALEDs and mTALEDs suggested that full-length DddA_tox_ E1347A could assist the ssDNA-specific TadA8e enzyme in accessing dsDNA [78]. Thus, the E1347A mutation in the DddA_tox_ protein does not seem to affect its ability to access dsDNA, although it significantly mitigates its intrinsic cytidine deaminase activity [39,40]. In a recent study, the crystal structures of DddA_tox_ E1347A, in complex with dsDNA substrates, were reported [80]. The structures showed that the mutant DddA_tox_ bound to the minor groove of dsDNA and caused it to bend sharply (~80 degrees away from the protein) [80]. This structural rearrangement significantly widened the minor groove of the dsDNA substrate, allowing direct base contacts with the active site residues [80]. In these observations, the Phenylalanine at 1375 intercalated into the dsDNA and displaced the 5′ thymine, which then replaced the target cytosine and formed a non-canonical T•G base pair with the juxtaposed guanine [80]. As a result of this series of events, the target cytosine within the 5′-TC motif was completely flipped out of the double helix, potentially allowing DddA_tox_ to locate it and deaminate it [80]. In the context of TALED design, the non-canonical T•G base pair could disrupt the canonical T•A base pair, thus allowing the adenine residue to be deaminated by TadA8e.

The single nucleotide sequence requirement inherent in the use of TadA8e means the potential off-target effects of TALED constructs will need to be critically evaluated before they can be utilized for human gene therapy. Whole mitochondrial genome sequencing detected notably low levels of off-target editing, at frequencies of 0.019% ± 0.002% for sTALEDs, 0.009% ± 0.001% for mTALEDs, and 0.008% ± 0.001% for dTALEDs [40]. With regard to nuclear off-target editing, targeted deep sequencing at a specific locus with high sequence homology to a sTALED target site did not reveal any significant nuclear off-target editing events [40]. However, a comprehensive evaluation of the nuclear genome-wide mutagenesis induced by TALEDs is still required.

## 3. Mitochondrial DNA Base Editing for Therapy and Modeling of Mitochondrial Diseases

Base editing techniques for correcting point mutations in nuclear DNA have been extensively used in animal models [38,81,82]. There has been significant progress in understanding the genetics of mitochondria and the connections between gene mutations and diseases [2,83]. Furthermore, acquired mtDNA mutations have been linked to aging, cancer, and neurodegenerative diseases [15,84,85,86]. With the rapid advancement of mtDNA editing tools and related science, it is expected that this innovative technique will soon be tested in clinical settings. DdCBEs and TALEDs allow for programmable C•G-to-T•A and A•T-to-G•C conversions in mtDNA, respectively, without DSBs [39,40]. As such, they hold the potential to model or correct pathogenic variants associated with mitochondrial disease and to increase our understanding of mitochondrial biology (Figure 2). An analysis of the MITOMAP database [13] (accessed on 15 January 2023) showed that 86 out of 91 confirmed point mutations in mtDNA associated with disease could potentially be corrected or modeled using DbCBEs or TALEDs (Figure 2) (Table 1).

## 4. Animal Models for Mitochondrial Dysfunction Using Mitochondrial DNA Base Editors

Animal models are valuable tools for understanding the underlying molecular mechanisms of disease progression and developing new treatments. Multiple research groups have created various gene edited animal models of mitochondrial diseases [9,87,88]. Most of these models were created by engineering various nuclearly encoded genes responsible for mitochondrial functions [87,89]. However, only a handful of animal models in which mtDNA has been engineered are currently available [23,89,90,91]. The vast majority of these models have been reviewed elsewhere [9,92], and a recently published review article extensively discussed the generation of animal disease models using DdCBEs [93]. In this section, we briefly summarized recent attempts to create animal models of mitochondrial disease using mitochondrial base editors.

Lee et al. [46] microinjected DdCBE-encoding mRNA into mouse embryos to introduce the pathogenic point mutation m.12918G>A, mimicking the m.13513G>A variant in human mtDNA. Previous studies confirmed that this mutation in the mouse mitochondrial *Nd5* gene was associated with multiple MDs, such as MELAS, LHON, and Leigh syndrome [94,95]. Additionally, they introduced the m.12336C>T nonsense mutation into the open reading frame of the mouse mitochondrial *Nd5* gene [46]. The injected embryos were implanted into surrogate mothers to generate new animal models with the potential to replicate human disorders [46]. A total of 4 out of 11 F0 mice had the m.12918G>A mutant allele, with a heteroplasmy level of 3.9–31.6%, and 9 out of 27 F0 mice had the m.12336C>T allele, with a heteroplasmy level of 0.22–57% [46]. The authors did not observe any clear phenotypes in newborn pups, possibly because they were too young or because a higher level of heteroplasmy was required for phenotypic translation [46]. However, this pioneering study demonstrated that induced mutations in mtDNA could be successfully transmitted to subsequent generations, and that animal models with mitochondrial dysfunction could be created using mitochondrial base editors [46]. Similarly, another group used DdCBEs to generate the m.12918G>A mouse model independently [96]. They generated the m.2820G>A mouse line, which mimicked the m.3376G>A mutation found in human mtDNA, associated with LHON and MELAS [96]. However, the authors did not report any phenotypic consequences resulting from these mutations [96].

A similar approach was used to create rat models of mitochondrial dysfunction [47]. Qi et al. [47] generated the m.7755G>A and m.14098G>A rat lines via DdCBEs to mimic the m.8363G>A and m.14710G>A mutations in human mtDNA, respectively, associated with distinct mitochondrial disorders [97,98]. The induced m.14098G>A mutation was transmittable and resulted in decreased ATP levels and Complex I activity in the hearts and brains of transgenic F1 animals [47]. In addition, mutation-harboring animals displayed a locomotive and cardiac phenotype, as assessed by open field test and echocardiography, respectively [47]. Moreover, no changes in protein expression levels were detected, suggesting that a higher level of heteroplasmy may be required to overcome the competitive advantage that wild-type mtDNA might display over variant mtDNA during protein translation [47].

Microinjection of DdCBE-encoding mRNA into zebrafish embryos was reported by two groups, including ours, to create various disease models, such as MELAS and LHON [48,49]. Shen lab [48] generated the m.4247G>A, m.14076G>A, and m.8892G>A zebrafish models, as orthologs to the human pathological m.3733G>A, m.13513G>A, and m.8363G>A mutations, respectively. The m.3733G>A mutation has been associated with LHON, the m.13513 G>A has been associated with MELAS or Leigh syndrome, and the m.8363G>A mutation has been associated with MERFF [94,97]. Mutations generated in the zebrafish model were maintained throughout development and successfully transmitted to subsequent generations [48]. The animal models for the m.3733G>A and m.13513 G>A mutations showed defective motility and abnormal mitochondrial morphology, as assessed by swim track assays and transmission electron microscopy, respectively [48]. Furthermore, our group generated a MELAS-like model with pleiotropic molecular consequences by introducing the m.3739G>A mutation in the zebrafish mitochondrial genome [49].

Animal models with knockouts of protein-coding genes in mtDNA are needed for the systematic and comprehensive investigation of mtDNA-related pathways. To this end, we previously developed TALE Writer, a computational tool for the efficient design of mitochondrial base editors, aimed at introducing premature termination codons in mitochondrial protein coding genes. This tool was tested in human and zebrafish mtDNA [49]. Recently, Minczuk lab [75] generated a library of mitochondrial base editors for the precise ablation of every protein-coding gene in mouse mtDNA, termed the MitoKO. In particular, this library comprised optimized G1333-split DdCBEs [75]. These base editors were used to generate nearly homoplasmic nonsense mutations (equivalent to gene knockout) in cell culture, with very precise on-target editing and background levels of off-target editing [75]. Additionally, using the MitoKO library, a mouse model with ATP synthase dysfunction was generated by introducing the m.8096G>A mutation in the *Atp6* gene, followed by selective breeding for several generations [75]. Patients with truncating *MT-ATP6* mutations had heterogeneous clinical symptoms, as the ATP6 protein is a subunit of Complex V, and suboptimal expression resulted in impaired mitochondrial function [99]. The molecular phenotypes observed in the m.8096G>A mouse model displayed a remarkable correlation with those in patients with truncating *MT-ATP6* mutations, demonstrating the clinical relevance of in vivo models of mtDNA dysfunction [75]. It is worth noting that induced heteroplasmy levels should be kept below a certain threshold to avoid potential lethality [75]. In the coming years, the MitoKO library is expected to be extensively used in biomedical research to understand the molecular mechanisms underlying mitochondrial dysfunction, and may facilitate the development of gene therapy for mitochondrial diseases.

Despite recent progress, options for generating animal models of mitochondrial diseases remain limited. Notably, the TALED system has not yet been used to create such models. One potential avenue for future research is the creation of conditional models with inducible, organ-specific mtDNA editing systems to better understand the etiology of mitochondrial diseases. Where rodent models do not accurately model human mitochondrial diseases, it may soon be possible to generate nonhuman primate models with mtDNA mutations that more closely resemble human conditions. This possibility is supported by recent reports of DdCBE-mediated base editing in 3PN human embryos by two different groups [51,52].

In particular, Chen et al. [51] used DdCBE-encoding mRNAs to introduce the m.3733G>A, m.8363G>A, and m.13513G>A mutations in 3PN human embryos, associated with LHON, MERRF, and MELAS or Leigh syndrome, respectively. High levels of on-target base editing for the m.3733G>A and m.8363G>A sites were observed, although editing at the m.13513G>A site was less successful [51]. As in the in vitro studies, bystander editing was detected around the target sites during testing in human 3PN embryos [51]. Moreover, a strong correlation between on-target and off-target editing was observed, suggesting that further optimization would be needed in order to minimize off-target editing before clinical use [51]. A similar approach was taken by Wei et al. [52], successfully introducing mutations at different target sites using the DdCBE platform. They observed that injections at the cleavage stage resulted in higher C-to-T editing compared to injections at the zygote stage [52]. The authors also noted the presence of off-target editing, emphasizing the requirement for additional optimization [52].

## 5. Conclusions and Future Perspectives

The groundbreaking development of DdCBEs [39], the first-ever technology for organellar base editing, marked a major milestone in the field of mitochondrial genome engineering and initiated a gene editing revolution. Indeed, canonical DdCBEs preceded other remarkable platforms for the precise manipulation of the mitochondrial genome, such as TALEDs [40], ZF-DdCBEs [73], and HiFi-DdCBEs [72], among others. Moreover, significant efforts have been made to improve the specificity of these novel tools. Broadly, these technologies have facilitated mtDNA editing in a variety of cell lines and animal models. Mitochondrial base editors have been successfully delivered via a number of strategies, including plasmid DNA lipofection, mRNA electroporation, and transduction with recombinant AAV particles. Overall, the mitochondrial genome engineering toolbox (Figure 3), which has expanded at an astounding pace, comprises notably reliable and versatile resources for targeted mtDNA de novo mutagenesis.

Precise editing of the human mitochondrial genome opens up the possibility of modeling mtDNA-based disorders, as well as developing gene therapies for a currently incurable set of diseases. Furthermore, the ability to make single edits in mtDNA could help improve our understanding of mitochondrial biology. For instance, introducing a premature stop codon to knock out a protein-coding gene could be of aid in elucidating the role of a specific mtDNA-related pathway on organismal homeostasis. To date, mtDNA base editing has been used to introduce a number of disease-associated variants in cultured human cell lines and animal models [46,47,48,49,63,75]. Remarkably, some of these strategies have resulted in expected phenotypic outcomes, such as impaired oxidative phosphorylation. Nonetheless, the utility of these engineered cellular and animal models for the elucidation of mtDNA-related molecular mechanisms or the development of gene therapies remains largely unexplored.

In addition to advances on targeting scope, activity, specificity, and applications of existing mitochondrial base editors, developing new tools would be beneficial. Similar to TALEDs, novel technologies that rely on the combined activity between full-length DddA_tox_ E1347A and other accessory effector domains could broaden the scope of possible mtDNA modifications. Metagenomic approaches could aid in discovering additional dsDNA-specific deaminases with unique properties. It is worth noting that current mtDNA editing technologies are limited by bystander editing. For example, targeting the most common human mtDNA pathogenic variant, m.3243A>G in the *MT-TL1* locus, remains challenging, given its sequence context, in which multiple bystander editing events are likely to occur. Thus, deaminase domains with unique sequence context preferences and more general strategies for the mitigation of bystander editing would significantly improve the applicability of mitochondrial base editors.

Thus far, advances in mtDNA base editing have remained focused on effector domain engineering, e.g., mutating DddA_tox_ or developing tandem deaminase domains. Similar to the ZF scaffold engineering efforts reported for ZF-DdCBEs, it would be beneficial to further characterize or advance TALEs in the context of mtDNA binding. For example, it remains to be determined whether mtDNA base editors containing TALEs that can recognize any base at the 5′ terminus of a binding site are as efficient and specific as their canonical counterparts. Furthermore, the effects of TALE length on the activity and specificity of TALE-based mtDNA base editors have yet to be thoroughly examined. In addition to ZF and TALE engineering, alternative all-protein DNA-binding moieties could be fused to dsDNA-specific effector domains, such as DddA_tox_ or other related molecules, to create alternative mtDNA base editors. These efforts would further expand the mitochondrial genome engineering toolbox.

Finally, the elucidation of a reproducible pathway for the import of nucleic acids into the mitochondrial matrix would enable further mtDNA manipulation, including CRISPR-based editing and other approaches. Such an advancement would significantly democratize the field of mitochondrial genome engineering, catalyzing the development of technologies for basic research, disease modeling, and gene therapy. In the meantime, all-protein technologies will remain at the forefront of the mtDNA editing revolution.

## Figures and Tables

**Table 1:** Pathogenic mitochondrial mutations confirmed with MITOMAP [13]. Out of 91 confirmed pathogenic point mutations, 86 can potentially be corrected or modeled using the DdCBE and TALED mtDNA base editing platforms. Figure 2 contains further details.

Locus	Allele SNP	Associated Disease	Treatment via C-to-T Editing	Modeling via C-to-T Editing	Treatment via A-to-G Editing	Modeling via A-to-G Editing
*MT-TF*	m.583G>A	MELAS/MM & EXIT		✓	✓	
	m.616T>C	Maternally inherited epilepsy/mito tubulointerstitial kidney disease (MITKD)/Gitelman-like syndrome	✓			✓
*MT-RNR1*	m.1494C>T	DEAF		✓	✓	
	m.1555A>G	DEAF; autism spectrum intellectual disability; possibly antiatherosclerotic	✓			✓
*MT-TV*	m.1606G>A	AMDF		✓	✓	
	m.1630A>G	MNGIE-like disease/MELAS	✓			✓
	m.1644G>A	Leigh Syndrome/HCM/MELAS		✓	✓	
*MT-TL1*	m.3243A>G	MELAS/Leigh Syndrome/DMDF/MIDD/SNHL/CPEO/MM/FSGS/ASD/Cardiac + multi-organ dysfunction	✓			✓
	m.3243A>T	MM/MELAS/SNHL/CPEO				
	m.3256C>T	MELAS; possible atherosclerosis risk		✓	✓	
	m.3258T>C	MELAS/Myopathy	✓			✓
	m.3260A>G	MMC/MELAS	✓			✓
	m.3271T>C	MELAS/DM	✓			✓
	m.3271delT	PEM/retinal dystrophy in MELAS				
	m.3280A>G	Myopathy	✓			✓
	m.3291T>C	MELAS/Myopathy/Deafness + Cognitive Impairment	✓			✓
	m.3302A>G	MM	✓			✓
	m.3303C>T	MMC		✓	✓	
*MT-ND1*	m.3376G>A	LHON MELAS overlap		✓	✓	
	m.3460G>A	LHON		✓	✓	
	m.3635G>A	LHON		✓	✓	
	m.3697G>A	MELAS/Leigh Syndrome/LDYT/BSN		✓	✓	
	m.3700G>A	LHON		✓	✓	
	m.3733G>A	LHON		✓	✓	
	m.3890G>A	Progressive Encephalomyopathy/Leigh Syndrome/Optic Atrophy		✓	✓	
	m.3902_3908 ACCTTGCinv	EXIT + myalgia/severe LA + cardiac/3-MGA aciduria/nephropathy + deafness + diabetes				
	m.4171C>A	LHON/Leigh-like phenotype				
*MT-TI*	m.4298G>A	CPEO/MS		✓	✓	
	m.4300A>G	MICM	✓			✓
	m.4308G>A	CPEO		✓	✓	
*MT-TQ*	m.4332G>A	Encephalopathy/MELAS		✓	✓	
*MT-TM*	m.4450G>A	Myopathy/MELAS/Leigh Syndrome		✓	✓	
*MT-TW*	m.5521G>A	Mitochondrial myopathy		✓	✓	
	m.5537_5537insT	Leigh Syndrome				
*MT-TA*	m.5650G>A	Myopathy		✓	✓	
*MT-TN*	m.5690A>G	CPEO + ptosis + proximal myopathy	✓			✓
	m.5703G>A	CPEO/MM		✓	✓	
	m.5728T>C	Multiorgan failure/ myopathy	✓			✓
*MT-CO1*	m.7445A>G	SNHL	✓			✓
*MT-TS1* precursor	m.7445A>G	SNHL	✓			✓
*MT-TS1*	m.7471_7472insC	PEM/AMDF/Motor neuron disease-like				
	m.7497G>A	MM/EXIT		✓	✓	
	m.7510T>C	SNHL	✓			✓
	m.7511T>C	SNHL/Deafness	✓			✓
*MT-TK*	m.8306T>C	Severe adult-onset multisymptom myopathy/ Myoclonic epilepsy	✓			✓
	m.8313G>A	MNGIE/Progressive mito cytopathy		✓	✓	
	m.8340G>A	Myopathy/Exercise Intolerance/Eye disease + SNHL		✓	✓	
	m.8344A>G	MERRF.; Other-LD/Depressive mood disorder/leukoencephalopathy/HiCM	✓			✓
	m.8356T>C	MERRF	✓			✓
	m.8363G>A	MICM+DEAF/MERRF/Autism/Leigh Syndrome/Ataxia + Lipomas		✓	✓	
*MT-ATP8/6*	m.8528T>C	Infantile cardiomyopathy/hyperammonemia	✓			✓
*MT-ATP6*	m.8851T>C	BSN/Leigh syndrome	✓			✓
	m.8969G>A	Mitochondrial myopathy, lactic acidosis and sideroblastic anemia (MLASA)/IgG nephropathy		✓	✓	
	m.8993T>C	NARP/Leigh Disease/MILS/other	✓			✓
	m.8993T>G	NARP/Leigh Disease/MILS/other				
	m.9035T>C	Ataxia syndromes	✓			✓
	m.9155A>G	MIDD, renal insufficiency	✓			✓
	m.9176T>C	FBSN/Leigh Disease/Spinocerebellar Ataxia	✓			✓
	m.9176T>G	Leigh Disease/Spastic Paraplegia/Spinocerebellar Ataxia				
	m.9185T>C	Leigh Disease/Ataxia syndromes/NARP-like disease/Episodic	✓			✓
		weakness and Charcot-Marie-Tooth				
	m.9191T>C	Leigh Disease	✓			✓
	m.9205_9206delTA	Encephalopathy/Seizures/Lacticacidemia				
*MT-TG*	m.10010T>C	PEM	✓			✓
*MT-ND3*	m.10158T>C	Leigh Disease/MELAS	✓			✓
	m.10191T>C	Leigh Disease/Leigh-like Disease/ESOC	✓			✓
	m.10197G>A	Leigh Disease/Dystonia/Stroke/LDYT		✓	✓	
*MT-ND4L*	m.10663T>C	LHON	✓			✓
*MT-ND4*	m.11777C>A	Leigh Disease				
	m.11778G>A	LHON/Progressive Dystonia		✓	✓	
*MT-TH*	m.12147G>A	MERRF-MELAS/Encephalopathy		✓	✓	
	m.12201T>C	Maternally inherited non-syndromic deafness	✓			✓
*MT-TS2*	m.12258C>A	DMDF/RP + SNHL				
*MT-TL2*	m.12276G>A	CPEO		✓	✓	
	m.12294G>A	CPEO/EXIT + Ophthalmoplegia		✓	✓	
	m.12315G>A	PEO/KSS/possible carotid atherosclerosis risk, trend toward myocardial infarction risk		✓	✓	
	m.12316G>A	CPEO		✓	✓	
*MT-ND5*	m.12706T>C	Leigh Disease	✓			✓
	m.13042G>A	Optic neuropathy/retinopathy/LD		✓	✓	
	m.13051G>A	LHON		✓	✓	
	m.13094T>C	Ataxia + PEO/MELAS, LD, LHON, myoclonus, fatigue	✓			✓
	m.13379A>G	LHON	✓			✓
	m.13513G>A	Leigh Disease/MELAS/LHON-MELAS Overlap Syndrome/negative association w Carotid Atherosclerosis		✓	✓	
	m.13514A>G	Leigh Disease/MELAS/Ca^2+^ downregulation	✓			✓
*MT-ND6*	m.14453G>A	MELAS/Leigh Disease		✓	✓	
	m.14459G>A	LDYT/Leigh Disease/dystonia/carotid atherosclerosis risk		✓	✓	
	m.14482C>A	LHON				
	m.14482C>G	LHON				
	m.14484T>C	LHON	✓			✓
	m.14487T>C	Dystonia/Leigh Disease/ataxia/ptosis/epilepsy	✓			✓
	m.14495A>G	LHON	✓			✓
	m.14568C>T	LHON		✓	✓	
*MT-TE*	m.14674T>C	Reversible COX deficiency myopathy	✓			✓
	m.14709T>C	MM + DMDF/Encephalomyopathy/Dementia + diabetes + ophthalmoplegia	✓			✓
	m.14710G>A	Encephalomyopathy + Retinopathy		✓	✓	
*MT-CYB*	m.14849T>C	EXIT/Septo-Optic Dysplasia	✓			✓
	m.15579A>G	Multisystem Disorder, EXIT	✓			✓
*MT-TP*	m.15990C>T	MM/PEO		✓	✓	
